# 1st Workshop of the Canadian Society for Virology

**DOI:** 10.3390/v9030054

**Published:** 2017-03-20

**Authors:** Craig McCormick, Nathalie Grandvaux

**Affiliations:** 1 Department of Microbiology and Immunology, Dalhousie University, 5850 College Street, Halifax, NS B3H 4R2, Canada; 2 Beatrice Hunter Cancer Research Institute, Dalhousie University, 5850 College Street, Halifax, NS B3H 4R2, Canada; 3 Département de Biochimie et Médecine Moléculaire, Université de Montréal, QC H3C 3J7, Canada; 4 Centre de Recherche du CHUM (CRCHUM), Montréal, QC H2X 0A9, Canada

**Keywords:** Canada, virology, oncolytic viruses, vaccines, antiviral drugs, pathogenesis

## Abstract

The 1st Workshop of the Canadian Society for Virology (CSV2016) was a Special Workshop of the 35th Annual Meeting for the American Society for Virology, held on 18 June 2016 on the beautiful Virginia Tech campus in Blacksburg, Virginia. The workshop provided a forum for discussion of recent advances in the field, in an informal setting conducive to interaction with colleagues. CSV2016 featured two internationally-renowned Canadian keynote speakers who discussed translational virology research; American Society for Virology President Grant McFadden (then from University of Florida, now relocated to Arizona State University) who presented his studies of oncolytic poxviruses, while Matthew Miller (McMaster University) reviewed the prospects for a universal influenza vaccine. The workshop also featured a variety of trainee oral and poster presentations, and a panel discussion on the topic of the future of the CSV and virus research in Canada.

## 1. Introduction

**Figure 1:**
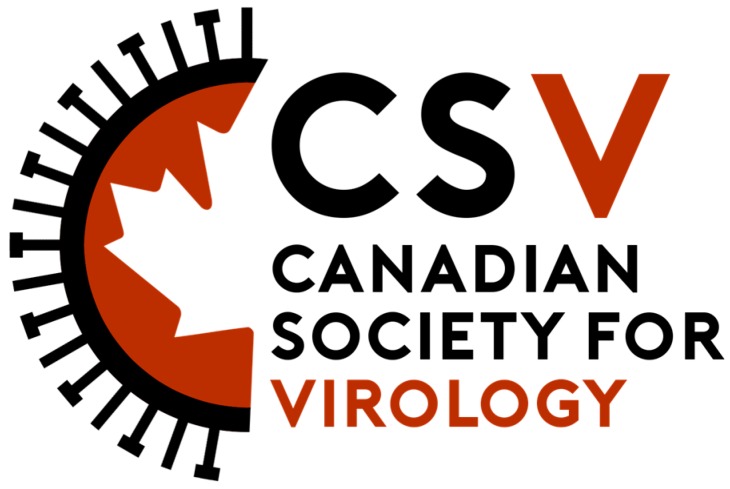
*Canadian Society for Virology* logo (art by Lucas Jarche, Dalhousie University, Halifax, NS, CA).

Canadian virologists, including world-renowned experts in basic, clinical and epidemiological research, have made important contributions to our fundamental understanding of many viruses, which has led to the development of new strategies for monitoring, preventing and treating viral diseases. For Canada to meet the challenge of emerging and chronic viral infections, and realize its potential to lead in this research area, the virology research community must come together to exchange ideas and discover new opportunities for collaboration. Recognizing the outstanding need for better ways for the Canadian virology research community to interact, we founded the Canadian Society for Virology (CSV)/Société Canadienne pour la Virologie (SCV) (Figure 1). We seek to create structures that will foster collaboration and accelerate the translation of virology research findings to positive health outcomes for Canadians. To launch the CSV, we developed a Special Workshop in conjunction with the 35th Annual Meeting for the American Society for Virology. This workshop was the first scientific activity of the newly-founded CSV. The objectives of the 1st Workshop of the Canadian Society for Virology (CSV2016) were:

(1) to highlight diversity and excellence of Canadian virology research;
(2) to mobilize Canadian virus researchers across different disciplines (basic, clinical, social and epidemiological) to create a broader base of research expertise committed to tackling new challenges.

## 2. Bunnies to Bone Marrow: ex vivo Virotherapy with Myxoma Virus

Following introductory comments from Dr. Nathalie Grandvaux (Université de Montréal and Centre de Recherche du Centre Hospitalier de l’Université de Montréal (CRCHUM)), the workshop opened with a keynote lecture from ex-patriate Canadian, Dr. Grant McFadden (then from University of Florida, now relocated to Arizona State University). In his role as American Society for Virology (ASV) president, Dr. McFadden welcomed CSV2016 attendees and expressed his support for the initiative. Well known as the leading expert on a rabbit poxvirus known as myxoma virus, McFadden has elucidated many aspects of virus–host interactions, linking key poxvirus proteins to immune evasion and pathogenesis [1]. He has also led efforts to test the oncolytic properties of these viruses. It is a widely-held view that defective antiviral defenses are an ‘Achilles Heel’ of cancer cells that can be exploited for oncolytic virotherapy. McFadden made the case for myxoma virus as a promising oncolytic agent; it can kill a variety of human cancer cells in vitro, it is nonpathogenic outside of the rabbit host, it is genetically tractable, and there is no evidence for pre-existing antibodies in the human population.

In recent years, McFadden has been investigating the potential of myxoma virus in ex vivo virotherapy to treat residual cancer. Autologous hematopoietic stem cell transplantation can repopulate the hematopoietic compartment following radiation and chemotherapy, but there is a risk that the autologous transplanted material could harbor cancer cells. McFadden showed that ex vivo myxoma virus therapy can delete cancer cells from transplants without harming self-renewing CD34^+^ stem cells [2]. This selectivity comes from the fact that myxoma virus can not bind or infect normal human CD34^+^ cells [3]. He also showed that ex vivo myxoma virus therapy can prevent one of the major adverse outcomes of allogeneic hematopoietic stem cell transplantation, known as graft vs. host disease (GVHD), which is caused by immunological attack on target recipient organs by donor allogeneic T cells [4,5]. McFadden closed his lecture with a tribute to renowned virologist Dr. John S. Colter, Chair of the Department of Biochemistry at the University of Alberta from 1961 to 1987, who hired McFadden into his first independent position and provided important early career mentorship.

## 3. Control of MAP Kinase Signal Transduction during Paramyxovirus Infection

The first trainee presentation was from M.Sc. student Alexa Robitaille (Université de Montréal and CRCHUM), who presented her studies of the regulation of antiviral signal transduction in response to paramyxovirus infection. Viral nucleic acids are recognized by sentinel proteins in the cytoplasm, including retinoic acid inducible gene I (RIG-I) and melanoma differentiation-associated protein 5 (MDA5) [6,7], which activate downstream signal transduction pathways that lead to the establishment of an antiviral state. The activation and termination of antiviral signal transduction is heavily regulated by post-translational modifications, most notably phosphorylation [8]. While many activating kinases have been identified, less is known about the phosphatases that negatively regulate these responses and limit the generation of inappropriate inflammatory responses. Most notably, no phosphatase has yet been identified that can dephosphorylate the p38 and Jun N-terminal kinase (JNK)/mitogen-activated protein kinase (MAPK) enzymes following paramyxovirus infection. Ms. Robitaille reported that dual specificity phosphatase 1 (DUSP1) is strongly upregulated in response to infection with the paramyxoviruses Sendai virus (SeV) or respiratory syncytial virus (RSV). Ectopic overexpression of DUSP1 during infection diminished the levels of phosphorylated p38 and JNK MAPKs, whereas DUSP1-specific RNA interference had the opposite effect. Surprisingly, DUSP1-mediated inhibition of MAPK phosphorylation had no effect on downstream phosphorylation of activating transcription factor 2 (ATF2)/c-Jun, or the levels of cytokines elicited during infection. Moreover, ectopic expression of DUSP1 has no effect on RSV replication in cell culture. Ms. Robitaille hypothesized that, in this system, DUSP1 may be affecting a different pool of p38 and JNK MAPK enzymes that govern alternative functions, while leaving activator protein 1 (AP-1)-mediated cytokine production intact.

## 4. Control of Hepatitis C Virus Replication by MicroRNAs

Hepatitis C virus (HCV) RNA is translated by host protein synthesis machinery into a long precursor polyprotein, which is processed into three structural and seven non-structural proteins that govern viral replication. A liver-specific microRNA (miRNA) known as miR-122 dictates HCV hepatotropism by binding directly to HCV RNA near the 5′-untranslated region and promoting HCV RNA translation and replication [9]. Argonaute (Ago) proteins are components of RNA-induced silencing complexes (RISC) that direct the activities of miRNAs, and are necessary for miR-122-mediated promotion of the HCV replication cycle [10]. Dr. Joyce Wilson from the University of Saskatchewan stepped in to present the research of her postdoctoral fellow Dr. Yalena Amador-Canizares, who was unable to present due to a travel delay. Dr. Wilson reported that they had used CRISPR/Cas9 genome editing technology to knock out Ago2, the only Ago protein with ‘slicing’ (RNA cleaving) activity. Ago2 knockout cells supported HCV replication, suggesting that at least one other Ago protein can sustain high levels of HCV replication in the absence of Ago2. Dr. Wilson indicated that it will be important to methodically knock out combinations of all four Ago proteins to further elucidate their role in HCV replication.

## 5. Development of an Alpaca Model for Middle East Respiratory Syndrome Coronavirus (MERS-CoV)

Camels are the primary amplifying host of MERS-CoV, and the source of outbreaks in the human population. A ‘One Health’ approach has been proposed to prevent human MERS-CoV infection, by vaccinating camels [11]. This camel model demonstrated efficient MERS-CoV replication in the upper respiratory tract and shedding of virus [12], but several factors, including high cost of obtaining and housing camels, limit the utility of this model for MERS-CoV vaccine development. Dr. Darryl Falzarano from the Vaccine and Infectious Disease Organization—International Vaccine Centre (VIDO-InterVac) presented a lower-cost alternative model in alpacas, a new world camelid. Previous work had demonstrated seropositivity for MERS-CoV in alpacas [13]. Furthermore, Dipeptidyl Peptidase 4 (DPP4), the cell surface receptor for MERS-CoV, is highly conserved between alpacas and camels, and alpaca kidney epithelial cells can be infected ex vivo [14]. Dr. Falzarano reported that MERS-CoV replicated to high titers in alpacas that were intranasally- and/or intratracheally-infected with MERS-CoV. Irrespective of route of administration, all animals had MERS-CoV RNA and infectious virus in nasal swabs, and seroconverted following infection, and there were no signs of clinical disease. Finally, Dr. Falzarano demonstrated that initial infection by the combined route offers protection from subsequent infection (as measured by viral RNA levels recovered from nasal swabs), suggesting that alpacas are a suitable alternative model for assessment of vaccine efficacy and prevention of virus shedding.

## 6. Monitoring and Visualizing RNA Virus Evolution

RNA virus replication generates complex ‘swarms’ of thousands of genetic variants with unknown and largely unpredictable properties. These high mutation rates are a major driver of RNA virus evolution, and the rapid emergence of adaptive mutations that affect tropism, virulence, fitness and drug resistance. Ex-patriate Canadian Dr. Marco Vignuzzi from the Pasteur Institute presented his approach to combine experimental evolution with mathematical modeling to determine the evolutionary trajectory of RNA virus mutants [15,16]. Deep sequencing data, combined with bioinformatics, permitted representation of mutations in 2D space, while fitness is represented in the third dimension. Using these approaches, Dr. Vignuzzi demonstrated that a virus’ sequence ‘space’ pre-determines its evolutionary trajectory, and altering this space can be used to attenuate viruses for vaccine development.

## 7. CD8 T Cell Responses that Promote Protective Immunity to HCV Infection

Approximately 25% of individuals with acute HCV infection can clear the virus spontaneously, but the immunological basis for protection from chronic HCV infection (and reinfections common amongst intravenous drug users) remains elusive. Indeed, some individuals fail to clear subsequent infections despite pre-existing HCV-specific memory T lymphocytes. As a graduate student at CRCHUM, under the mentorship of Dr. Naglaa Shoukry, Dr. Mohamed Abdel-Hakeem demonstrated that viral persistence upon HCV reinfection was associated with limited expansion of virus-specific T cells, whereas protection correlated with expansion of broad sets of HCV-specific T lymphocytes [17]. By conducting longitudinal T cell receptor analysis and creating and characterizing CD8 T cell clones from sorted cells, Dr. Abdel-Hakeem discovered that protective immunity upon HCV reinfection was associated with focusing of the repertoire of HCV-specific CD8 T cells recruited from the memory pool whereby clonotypes with the highest functional avidity were selected [18]. These studies have implications for the rational design of HCV vaccines.

## 8. Molecular Mechanisms of HIV Suppression of Antigen Presentation

The human immunodeficiency virus type 1 (HIV-1) prevents efficient recognition of infected cells by cytotoxic T lymphocytes (CTLs). The HIV-1 Nef protein contributes to immune evasion by blocking the cell surface localization of major histocompatibility complex I (MHC-I), thereby preventing the presentation of viral antigens. The molecular mechanisms of Nef-mediated alterations in protein trafficking remain incompletely understood. Using bimolecular fluorescence complementation (BiFC) to detect interactions between Nef and MHC-I, Brennan Dirk (Western University) reported that Nef/MHC-I complexes localize to Rab5-positive early endosomes and the trans-Golgi network, but not to the lysosomal compartment [19,20]. Site-directed mutagenesis revealed that residues Y320 and D327 in the carboxy-terminal tail of MHC-I were essential to maintain complex formation with Nef. These studies reveal that Nef interacts with the cytosolic tail of MHC-I to divert it from recycling endosomes to Rab5-positive early endosomes, highlighting the importance of the early endocytic network in the removal of MHC-I from the surface of infected cells.

## 9. Host-Targeted Antiviral Drug Discovery for Ebola Virus

In the last talk of the morning session, Corina Warkentin (University of Ottawa) presented her discovery of small molecules that block the entry of Ebola virus (EboV) into human cells. Successful EboV infection requires macropinocytosis of virions from the cell surface and trafficking to the late endosome or lysosomal compartments for interaction with its receptor known as the Niemann-Pick C1 protein (NPC1) [21,22,23,24]. Using pseudotyped murine leukemia viruses (MLVs) bearing EboV glycoproteins to screen for small molecules that could selectively block infection, led to the identification of several molecules that function in the low micromolar range. Ms. Warkentin presented her findings that a lead molecule, Compound 1.0, blocked infection of native EboV, as well as a variety of MLVs pseudotyped with entry glycoproteins from Lassa fever virus, lymphocytic choriomeningitis virus, Junin virus, and severe acute respiratory syndrome coronavirus (SARS-CoV). The related filovirus, Marburgvirus, was resistant to Compound 1.0. Virus attachment was unperturbed, suggesting that Compound 1.0 might be targeting a common host entry factor required for a post-attachment step.

## 10. Broadly-Neutralizing Antibodies against Influenza A Virus: The “Universal” Importance of the Polyclonal Response

Following the lunchtime poster session, Dr. Matthew Miller (McMaster University) delivered the 2nd keynote presentation of the day, on the topic of broadly-neutralizing antibodies (bnAbs) and future prospects for a universal influenza vaccine. Dr. Miller described the limitations of current multivalent seasonal vaccines and the monovalent 2009 H1N1 pandemic vaccine that had negligible effect on the spread of virus due to the rate of vaccine production and distribution. These factors have necessitated new approaches to vaccine development. The discovery of bnAbs that target the highly-conserved stalk domain of hemagglutinin have raised hopes for vaccines capable of conferring “universal” immunity against influenza viruses. These antibodies are normally made in low quantities, but can be substantially boosted through exposure to “pandemic-like” hemagglutinin (HA) proteins [25,26,27], and by overcoming the immunodominance of HA head domains [28]. By focusing research efforts on natural, polyclonal responses, Dr. Miller has discovered that both antibody isotype and specificity have major consequences on bnAb function. Specifically, stalk-binding antibodies of IgA isotype have a greater capacity to neutralize virus [29], and Fc-dependent effector functions of bnAbs are regulated by interactions among multiple antibody specificities [30]. These findings are likely to have profound implications for the successful generation of “universal” influenza virus vaccines and therapeutics.

## 11. Driving Forces for Aleutian Mink Disease Virus Evolution

For 40 years Aleutian mink disease virus (AMDV) was the sole species in the genus *Amdoparvovirus*. Recent advances in DNA sequencing technology led to the identification of four novel *Amdoparvoviral* species, igniting new studies of molecular pathogenesis and epidemiology [31,32]. Dr. Marta Canuti (Memorial University of Newfoundland) presented studies of AMDV molecular epidemiology in farmed and wild animals in Newfoundland, and contrasted this data with worldwide AMDV phylogeography and evolution. AMDV was highly prevalent in Newfoundland farmed and wild mink, with high diversity within single farms, high co-infection rates, farm-specific recombinants and polymorphisms within single infected individuals. Finally, the predominance of negative selection pressure on AMDV proteins was observed [33]. Dr. Canuti documented the movement of viruses between farms and the wild but also the existence of a wild-specific lineage. Globally, viruses from different countries fell within the same clades but formed country-specific subclades. These studies indicate that high AMDV prevalence in farms facilitates co-infections that favor viral recombination, further increasing viral diversity. AMDV moves from farms to the wild and is exchanged between different farms and countries, with subsequent evolution of parallel lineages generated by rapidly evolving viruses.

## 12. Eliciting Neutralizing Antibodies for HCV Vaccine Development

A prophylactic vaccine covering all seven major genotypes (gts) of HCV remains elusive. Dr. Jason Wong (University of Alberta) reported on efforts to develop an effective HCV vaccine that elicits bnAbs that bind conserved regions on HCV envelope glycoproteins critical for virus entry, known as E1 and E2 (E1E2). An E1E2 vaccine derived from a single gt1a strain (HCV-1) has been shown to elicit bnAbs in guinea pigs, chimpanzees, goats, and healthy human volunteers [34,35,36]. Epitope mapping studies revealed that immunized goat and human antisera competed with bnAb binding, and competed especially well with bnAbs known to block the interaction between E2 and the major cell entry receptor CD81 [36]. Indeed, HCV-1 E1E2-based vaccines directly blocked the E2-CD81 interaction. Collectively, these studies suggest that immunizing with HCV envelope glycoproteins elicits nAbs that block early stages of HCV entry. These results support the use of such a vaccine antigen to induce cross-genotype neutralization. 

## 13. Conclusion

**Figure 2:**
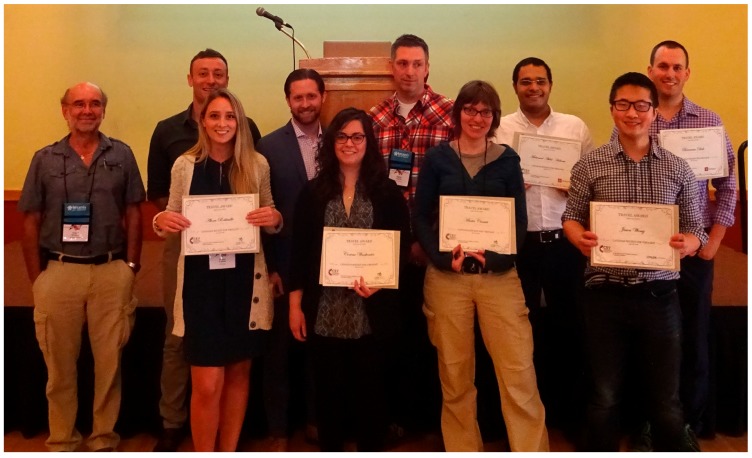
Presentation of travel awards to trainee presenters at the 1st Workshop of the Canadian Society for Virology (CSV2016). Each trainee abstract selected for an oral presentation by an arm’s-length peer review committee received a travel award. Also pictured are principal investigators that gave oral presentations. Front row: Grant McFadden (then University of Florida, Gainesville, FL, USA, now Arizona State University, Tempe, AZ, USA), Alexa Robitaille (University of Montreal Hospital Research Centre—CRCHUM—Montreal, QC, CA), Corina Warkentin (University of Ottawa—uOttawa—Ottawa, ON, CA), Marta Canuti (Memorial University—MUN—St. John’s, NL, CA), Jason Wong (University of Alberta, Edmonton, AB, CA). Back row: Marco Vignuzzi (Pasteur Institute, Paris, FR), Matthew Miller (McMaster University, Hamilton, ON, CA), Darryl Falzarano (Vaccine and Infectious Disease Organization-International Vaccine Centre—VIDO-InterVac—Saskatoon, SK, CA), Mohamed Abdel-Hakeem (CRCHUM, University of Pennsylvania—UPenn—Philadelphia, PA, USA), Brennan Dirk (Western University, London, ON, CA).

The CSV2016 Workshop closed with a group discussion led by Dr. Craig McCormick, concerning CSV priorities and future activities. There was consensus that the CSV should play an integral role in the development of a strong, multidisciplinary community of virus researchers in Canada. There was agreement that the primary focus of the CSV should be to organize recurrent workshops, which should be held independently, or in conjunction with other meetings as opportunities arise. There was strong support for the idea that future CSV workshops should maintain a focus on trainees, by keeping costs low to maximize opportunities for trainee attendance, by providing ample opportunities for oral and poster presentations, and by providing travel awards for selected peer-reviewed abstracts (Figure 2). Some urged collaboration with the well-established Canadian Society for Microbiology (CSM) on common goals. This discussion strengthened the foundation of the CSV and left the community with high expectations for upcoming CSV activities. Following this discussion, CSV2016 adjourned, and the group joined their colleagues from around the world at the ASV2016 reception and opening keynote lecture.
